# Hyaluronidase Use in Aesthetic Medicine: Formulations, Safety, and Clinical Practice

**DOI:** 10.3390/jcm15010279

**Published:** 2025-12-30

**Authors:** Francesca Arrigoni, Stefania Belletti, Silvia Caboni, Maurizio Cavallini, Andrea Cordovana, Riccardo Lazzari, Marco Francesco Papagni

**Affiliations:** 1 Agorà—Italian Society of Aesthetic Medicine, 20122 Milan, Italy; 2 Focus Group “Complication”, Agorà—Italian Society of Aesthetic Medicine, 20122 Milan, Italy; sbellez@libero.it (S.B.); silvicab@gmail.com (S.C.); maurizio.cavallini@libero.it (M.C.); dott.cordovana@gmail.com (A.C.); dr.riccardolazzari@gmail.com (R.L.); dottmarcopapagni@gmail.com (M.F.P.)

**Keywords:** hyaluronic acid, dermal filler, aesthetic procedure, cosmetic medicine, complication

## Abstract

The rising number of Hyaluronic acid (HA) filler applications has led to an increase in the incidence of complications, which can range from undesired aesthetic outcomes to severe vascular occlusion events. Hyaluronidase (Hyal) hydrolyzes HA molecules and represents the preferred treatment for managing complications associated with HA fillers. Although Hyal formulations are safe and effective, Hyal use for treating complications of aesthetic procedures remains off-label in many countries due to the lack of standardized protocols and varying recommendations on dosing strategies. Here, we review the use of Hyal in aesthetic medicine, focusing on the available formulations, including galenic preparations, and the associated risks. We provide an update on the current clinical practice for Hyal administration by reviewing cases reported in the literature from 2020 to 2025. We summarize the current dosage strategies and administration techniques for treating different complications, with details on newly developed protocols, the implementation of imaging guidance, and adjunctive treatments. Despite the great variability in dosage and protocols, Hyal administration is a safe and effective treatment to manage complications and undesired aesthetic outcomes caused by HA fillers. Future efforts should focus on developing standardized protocols to facilitate the decision-making process, reduce response time, and ensure successful outcomes.

## 1. Introduction

The demand for aesthetic and rejuvenation procedures has increased significantly over the past two decades, with hyaluronic acid (HA) being the most popular dermal filler, thanks to its unique properties [1,2]. HA is an optimal candidate for providing hydration, support, and augmentation to tissues like the skin. Being a natural compound, HA is minimally immunogenic, and it is naturally reabsorbed by the body after an average of 12 months, significantly reducing adverse events such as inflammatory reactions [2,3]. However, the rising number of aesthetic interventions led to an increasing occurrence of adverse events, ranging from swelling and filler misplacement to more serious complications, such as nodules, hypersensitivity reactions, or vascular occlusion [1,4].

Hyaluronidases (Hyals), a family of enzymes that hydrolyze HA molecules, are essential for managing corrections of undesired aesthetic outcomes and complications from filler procedures, enabling practitioners to dissolve HA in a controlled manner [5]. Although Hyal is widely used in clinical practice, as highlighted by the increasing number of case reports in the literature, a lack of standardized protocols and official guidelines concerning Hyal formulations, dosing, and administration methods persists [3,6]. In fact, recent literature highlights considerable heterogeneity in Hyal administration protocols, including decisions regarding dosage selection, injection techniques, adjuvant treatments, and allergy pre-testing. As a result, the absence of standardized protocols forces practitioners to determine treatment strategies on an individual basis in their daily clinical practice. This can delay effective treatment, undermine practitioners’ confidence, affect patient trust, and result in inconsistent outcomes. Additionally, due to the lack of official guidelines, Hyal use for cosmetic correction is still not officially approved by the European Medicines Agency (EMA); however, Hyal off-label use is widely recognized [2,7].

This review provides a comprehensive overview of the use of various Hyal formulations in aesthetic medicine, including galenic Hyal preparations, and discusses the current practice regarding allergy pre-testing. Finally, considering the rising number of cases documented in the literature, this review summarizes Hyal use in cosmetic medicine over the past five years, providing an updated overview of current treatment strategies and recent advancements in Hyal administration based on specific filler-related complications. Ultimately, this review highlights the significant variability of protocols used in clinical practice, emphasizing the need for the development of standardized guidelines.

## 2. Methods

A literature search was performed on PubMed using the keywords: “hyaluronidase”, “cosmetic”, “aesthetic”, “beauty”, “hypercorrection”, “complication”, “adverse”, and combinations thereof. No stringent inclusion or exclusion criteria were adopted. Articles were selected based on their relevance to the main topic of the manuscript, with particular attention to recent studies. Studies reporting cases of Hyal use for cosmetic correction between 2020 and 30 November 2025 were selected using the PubMed search: “hyaluronidase AND (esthetic OR aesthetic OR cosmetic OR beauty) AND (Hypercorrection OR complic* OR adverse)”. Inclusion criteria were (i) the Hyal administration protocol was described, (ii) Hyal was administered to treat complications caused by HA-based fillers, and (iii) Hyal injections represented the main treatment strategy. Due to the large number of studies present in the literature, representative articles documenting the heterogeneity of Hyal administration strategies were selected (n = 86) and have been included in Table S1.

## 3. Hyaluronidase Characteristics and Formulations

Hyal enzymes degrade HA polymers, thus temporarily reducing tissue viscosity and permeability. Due to these characteristics, Hyal is widely used in medicine to improve drug absorption and facilitate local anesthesia, as well as for off-label HA-filler degradation in cosmetic medicine [3,5]. Hyals are common molecules found in various biological sources. The human genome contains six genes, which encode HYAL1-4, HYAL-6, and PH-20. Hyal genes are also found in mice and in ovine and bovine species [8]. Additionally, Hyal has been identified in leeches, in the venom of bees and snakes, and in several microorganisms [5]. Hyals from mammalian origin and in bee venom degrade HA by cleaving β-1,4 glycosidic bonds, while leech Hyals cleave β-1,3 glycosidic bonds. Microbial Hyals cause β-elimination at β-1,4 glycosidic bonds, generating unsaturated disaccharides [8,9]. Although the molecular structure of different Hyals may vary, the catalytic structural domain, responsible for their function, is conserved in their predicted 3D structures [5,10]. Hyals of mammalian origin are the most employed, as they share over 20% sequence similarity with human Hyals; however, they may cause hypersensitivity reactions [5]. Recently, recombinant human Hyal (RHH) has been developed, offering higher purity than animal-derived Hyals, thereby reducing the risk of allergic reactions [3]. However, RHH availability remains limited to a few countries, while Hyals of mammalian origin are widely used worldwide. While the FDA has approved RHH (Hylenex^TM^) and animal-derived Hyal formulations such as Amphadase^TM^, Hydase^TM^, Vitrase^®^, and Hyalase^®^, Hyal use in aesthetic medicine in other regions, including Canada and the European Union (EU), remains off-label due to a lack of systemic assessments [2,6,11]. Animal-derived Hylase^®^ Dessau is the only injectable excipient-free formulation approved for anesthesia procedures within the EU, specifically in Germany [6]. Additionally, practitioners should be aware that different Hyal formulations contain varying amounts of active enzyme. For instance, Hylenex^TM^ and Vitrase^®^ contain 150 and 200 IU of Hyal, respectively, while other formulations like Hyalase^®^ have a higher Hyal concentration of 1500 IU [6]. Italy currently offers injectable Hyal only as galenic formulations, which can be liquid or lyophilized [12,13]. Italian aesthetic practitioners have recommended reconstituting Hyal with saline solution at different final concentrations, depending on the cosmetic complications. According to national regulations, galenic formulations should be used within six months of manufacture [13]. Similarly, over 23% of Canadian practitioners use galenic Hyal formulations for treating aesthetic complications [11].

## 4. Risk and Prevention of Adverse Reactions to Hyaluronidase

Although rare, allergic reactions to Hyal injections can occur, with an estimated rate of 0.05–0.7%, while most side effects are reported during ophthalmic procedures [8]. Hyal administration route and dosage are considered critical factors in the occurrence of adverse events that are not linked to hypersensitivity reactions [8,14]. Localized injections of high-dose Hyal can trigger erythema, inflammation, and swelling, while high-dose Hyal administered intravenously has been linked to more generalized symptoms [3,14].

Most allergic reactions are immediate hypersensitivity reactions (type I, immunoglobulin E-mediated), occurring within few minutes to hours after the procedure, but delayed hypersensitivity reactions (type IV) can also occur several hours to days after Hyal administration [8]. Delayed type IV response may occur in subjects who have not been previously treated with Hyal, while type I reactions can happen within minutes in subjects who are naïve to Hyal injections, but have been sensitized by cross-reactive agents [14]. Hyal formulation is also considered a potential cause of allergic reactions, with protein impurities and reconstitution substances used for lyophilized Hyal being possible sources of allergies; however, no current studies indicate a link to these substances [15]. Due to the high sequence similarity among mammalian Hyals, various protocols for the purification of Hyal from ovine and bovine sources are being explored to enhance enzyme purity while maintaining adequate enzyme activity [16]. Overall, RHH is addressed as the purest Hyal formulation with the highest activity per total enzyme milligram [3]. Indeed, while 5% of the general population carries RHH-reactive antibodies, there have been no reports of adverse events or allergic reactions to RHH alone [17].

The presence of preformed IgE antibodies against Hyal epitopes is the primary cause of allergic reactions to Hyal administration. For instance, IgE antibodies were identified in oncological patients who developed anaphylactic reactions to intravenously administered bovine-derived Hyal [18]. Interestingly, hymenoptera venom contains Hyal as a spreading substance; therefore, subjects allergic to these insects have a great risk of developing allergic reactions to Hyal injections, and those who are sensitized to the venom face a high risk of severe reactions due to IgE cross-reactivity [14,15]. A recent study evaluated the allergenicity of different hyaluronidases from bees and wasps. The Hyal present in honey bee venom was identified as a key allergen, able to activate basophils, strongly sensitizing susceptible patients, compared to Hyals from paper wasp and yellow jacket venoms [19]. Interestingly, a study investigating sensitization to Hyal in patients with a history of hypersensitivity reactions to hymenoptera venom found that subjects allergic to wasps are at higher risk of developing allergic reactions to Hyal administrations. Whereas, non-allergic individuals and those who received previous venom immunotherapy showed no reaction to Hyal administration [20]. As Hyal antigenicity is determined by its epitope structures, a B-cell epitope of Hyal from honey bee venom has recently been identified. This represents progress in developing targeted immunotherapy for allergic individuals, reducing the risk of hypersensitivity reactions when exposed to hymenoptera venom or during medical Hyal treatments [21].

Besides people allergic to hymenoptera venom, certain individuals are more likely to develop more severe anaphylaxis when they experience a type I response, such as those on ACE inhibitors or beta blockers, and those with hereditary angioedema and mast cell disorder, because it will be more difficult to restore hemodynamic stability during the systemic allergic reaction [14].

Currently, conflicting indications exist in the literature concerning the use of skin prick tests and allergic intradermal tests (IDT). Among the cases reported in the last five years, only two studies performed skin tests before proceeding with localized high-dose injections using animal-derived Hyal [22,23]. A recent survey investigating over 200 Australian practitioners showed that only 26% always performed skin tests, while most practitioners performed them occasionally [6]. The recent Italian consensus recommends avoiding allergy tests due to the lack of validated guidelines for their performance and to prevent misinterpretation, which can arise when testing is conducted by practitioners without the necessary expertise [13]. For instance, IDT provides results in 20 min, likely giving false-negative results in cases of type IV reactions [8]; however, a positive delayed reading after 48 or 72 h could suggest a T-mediated sensitization. Overall, experts have recently advised against routine testing in patients without a history of hypersensitivity reactions due to the lack of validation of skin testing. In particular, practitioners should refrain from performing skin testing in subjects with a history of anaphylaxis to Hyal or hymenoptera venom. Instead, these patients should be referred to an immunologist or to an allergy clinic, where tests can be performed safely with adequate anaphylaxis support and properly interpreted [14,24]. However, if the practitioners decide to perform an IDT, the indications from the Complications in Medical Aesthetic Collaborative organization should be followed, using 15 IU of animal-derived or galenic Hyal, although no validated Hyal concentration is currently defined for IDT [14,25]. Additionally, before Hyal administration, a thorough investigation of the patient’s medical history and previous allergic reactions should be conducted [14].

## 5. Complications of Hyaluronic Acid Filler Injections

### 5.1. Suboptimal Aesthetic Outcomes

HA filler not properly placed can result in asymmetries, overfilling, or the so-called Tyndall effect [26]. The Tyndall effect refers to a bluish-grey discoloration beneath the skin, caused by light scattering through particulate filler positioned too superficially, especially in areas with thin skin, such as the tear trough. It usually lasts for long periods of time and requires firm massages and Hyal interventions [3]. Asymmetries, lumps, and contour irregularities typically occur if the filler is not properly distributed or excessively administered, leading to an unnatural appearance and patient dissatisfaction [3]. Hyal can be administered to correct these outcomes and ensure proper cosmetic interventions in a second instance [27,28].

### 5.2. Structural Complications

Structural complications include edema, bruising, and temporary swelling, typically appearing immediately after the procedure, or localized lumps and non-inflammatory nodules, which are usually painless [1,26]. These complications may arise due to poor injection techniques, overcorrection, or improper filler selection for the treated area, and can lead to filler accumulation, misplacement, or migration. Importantly, complications due to filler migration may occur even after long periods of time from the procedure [1,29].

### 5.3. Inflammatory and Immunologic Complications

Inflammatory and immunologic complications include the development of inflammatory delayed-onset nodules (DON), which can appear several weeks after filler injection. Inflammatory DON are typically red, warm, and painful and are among the most common complications from HA filler procedures [3]. DON can develop following a delayed hypersensitivity reaction to filler material or to inflammatory HA fragments produced after filler degradation, or as a response to biofilm formed on the filler material by bacteria introduced during the injection. Infection can also occur due to bacterial proliferation at injection site [26,30]. Foreign body granulomas can develop several months after HA injection and appear as cystic formations composed of immune cells due to failed phagocytosis of foreign substances, leading to chronic inflammation [1,3].

Although rare, persistent edema and allergic responses may also occur, with reports of both type I and IV reactions, resulting in angioedema or anaphylaxis and erythematous swelling, respectively. The mechanisms triggering these reactions are not fully understood. Overall, HA is not considered a significant allergen, being naturally present in the human body; however, low-molecular-weight HA may cause hypersensitivity, and protein impurities and additives used during filler preparation may trigger allergic reactions [1,31]. Interestingly, delayed hypersensitivity reactions to HA filler have been reported following influenza-like infections and even COVID-19 vaccines [32,33].

### 5.4. Vascular Complications

Vascular damage and occlusion are severe complications of filler procedures, with an incidence rate between 0.001% and 0.005% [34], and can result from different mechanisms. Vessels can be damaged during the injection, leading to bleeding, swelling, and inflammation [4]. Vascular occlusion can occur due to extravascular compression, when filler material directly presses against blood vessels, or due to tissue swelling as HA retains water. Occlusion caused by intravascular embolism occurs when filler is injected within a vessel [4,22]. Vascular occlusion is characterized by a blockage of blood flow, which reduces or interrupts oxygen supply. Early signs include pain, changes in skin color (blanching and livedo patterns), and delayed capillary refill [3]. If not promptly treated, vascular impairment can progress. Eventually, tissue ischemia and necrosis will occur, with risks of infection and scarring [4]. Vascular ischemia is usually localized; however, when the damage is extensive or high-pressure injection techniques are used, occlusion can occur farther from the treated area [34]. Venous occlusion can result in necrosis and pulmonary embolism, whereas arterial occlusion can cause tissue necrosis and, depending on the artery affected by the retrograde embolic movement, stroke or blindness may occur [4]. Notably, permanent vision loss is a devastating consequence of filler injection and occurs due to HA emboli, which, traveling retrograde, can reach and block the central retinal artery [4]. Filler injection in areas surrounding the eyes, such as the glabella, forehead, and nasal dorsum are at higher risk of developing visual compromise, as they are rich in vessels connected to the ophthalmic artery [35,36,37,38,39,40].

## 6. Hyaluronidase for the Management of Filler Complications

### 6.1. General Considerations for Hyaluronidase Dosage and Concomitant Treatments

Hyal dosages and administration techniques vary depending on the type of complication, the anatomical location, and the filler characteristics.

Although standardized protocols are missing, general dosage ranges are outlined based on published reports and expert experiences [3,41]. For instance, localized lumps and aesthetic corrections are usually treated with low-dose Hyal injections, in a range between 5 and 150 IU, while vascular complications typically require higher doses, typically ranging from 300 to 1500 IU at regular intervals for up to four sessions [3,41], as has been confirmed in recent case reports [42,43,44,45]. The anatomical location also affects the required Hyal dosage. Delicate facial areas with thin skin may need low-dose Hyal. For instance, 3 IU injections were used to treat delayed lower eyelid edema caused by HA filler in the supraorbital area [46]. The Aesthetic Complications Expert (ACE) Group outlined a guide for Hyal administration based on facial regions. According to the ACE Group, 15–30 IU of Hyal are necessary to treat the nasal and perioral region, 10–15 IU for the infraorbital region, 3–4.5 IU for the periorbital region, and 1.5 IU for the lower lid area [41]. However, despite reviews and expert experience being available in the literature, the dosages and administration strategies used in clinical practice remain highly heterogeneous and are based on practitioners’ individual evaluations and preferences.

In their decision-making, practitioners should also consider the Hyal formulation used, since they vary in enzyme concentration [6]. Consequently, high-dose treatments with formulations containing low Hyal concentrations require the injection of multiple vials, increasing the risk of injection-related complications.

Overall, identifying the specific HA formulation is critical to establish an effective protocol, as the filler cross-linking degree greatly impacts its dissolution rate [47]. Indeed, newly highly cross-linked fillers are characterized by strong hydrogen bonds, making the filler dense, thus requiring high Hyal doses or multiple administrations [48]. Depending on the aesthetic purpose, monophasic or biphasic fillers can be used. Monophasic fillers are dense, uniform gels with a higher degree of cross-linking, while biphasic fillers have a granular structure, as cross-linked HA particles are suspended in a non-cross-linked carrier matrix [48,49]. In vivo and in vitro experiments have been conducted to assess Hyal efficacy for different filler types and found that degradation of monophasic fillers requires higher Hyal doses and longer exposure time compared to biphasic fillers [50]. Consequently, although the general consensus is that 50–150 IU of Hyal are sufficient to dissolve 1 mL of HA filler, modern highly cross-linked monophasic fillers may require 600–750 IU Hyal per milliliter of filler [48,51]. Moreover, for the establishment of a Hyal protocol, practitioners need to be aware of the reaction time of different HA fillers to Hyal. Generally, biphasic fillers can be dissolved quickly, from 5 min up to 1–2 h, while monophasic fillers may require up to 24 h [48]. Consequently, high Hyal doses are preferred in emergency scenarios involving vascular occlusion, to reduce the filler dissolution time. For non-emergent aesthetic corrections, a lower Hyal dose can be employed to manage the complications without affecting the filler’s augmentation effect. Interestingly, ultrasound analysis monitoring filler dissolution demonstrated that the first hour after Hyal administration registered the greatest volume reduction, independent of the characteristics of the fillers analyzed [52]. However, as Hyal activity is estimated to reduce after 30 min and last for around six hours, multiple interventions may be necessary to treat persistent filler masses [48,50].

Along Hyal injections, antibiotics and corticosteroids are often administered either locally or systemically to manage infection, inflammation, and allergic reactions [13]. Although these therapies are frequently used as first-line treatment, they are usually not sufficient to resolve the symptoms, as filler material persists, requiring Hyal administration [53,54,55]. In most studies, antibiotics and corticosteroids are administered either concurrently or following Hyal treatment, or after surgical interventions [35,37,56,57]. Surgical interventions to remove filler and inflammatory material may be necessary if symptoms persist [57,58,59]. Recently, fractional CO_2_ laser and fractionated radiofrequency (RF) microneedling have been implemented following Hyal administration in cases of skin necrosis and edema to promote tissue contraction, filler dissolution, skin healing, and minimize scar formation [60,61]. Hyperbaric oxygenation treatment (HBOT) is also frequently used as a coadjuvant treatment for managing vascular complications to restore blood flow and accelerate healing [56].

**Figure 1:**
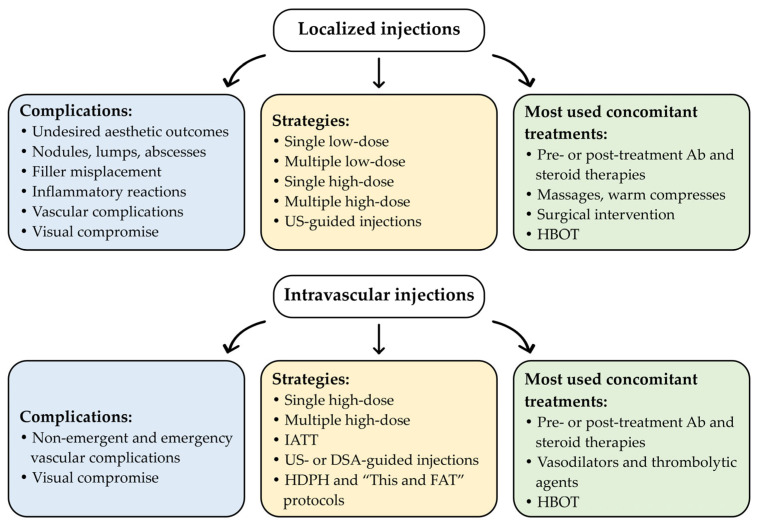
Schematic overview of the current Hyal administration strategies used in aesthetic clinical practice, based on case reports published over the past five years. A comprehensive overview is provided in Table S1. Abbreviations: Ab, antibiotic therapy; DSA, digital subtraction angiography; HBOT, hyperbaric oxygen therapy; HDPH, high-dose pulsed Hyal technique; IATT, intra-arterial thrombolytic therapy; “This and FAT”, T: botulinum toxin type A, h: high-dose HYAL, i: injectable platelet-rich fibrin, s: serum platelet-rich fibrin, a: aspirin and antibiotics, n: nanofat, d: debridement and dermabrasion, FAT: fat membrane application; US, ultrasound.

Overall, due to the absence of standardized protocols, great variability in Hyal administration protocols remains. Figure 1 and Table S1 summarize the different management strategies for Hyal administration currently used in clinical practice, including the implementation of newly developed protocols and concomitant treatments, based on the case reports documented in the literature over the past five years [12,22,23,28,29,32,33,34,35,36,37,38,39,40,42,43,44,45,46,53,54,55,56,57,58,59,60,61,62,63,64,65,66,67,68,69,70,71,72,73,74,75,76,77,78,79,80,81,82,83,84,85,86,87,88,89,90,91,92,93,94,95,96,97,98,99,100,101,102,103,104,105,106,107,108,109,110,111,112,113,114,115,116,117,118,119].

The following paragraphs describe the main strategies currently used for Hyal administration, based on recent case reports from the literature, with the aim of emphasizing the high variability of Hyal protocols and highlighting the need to develop standardized protocols and recommendations.

### 6.2. Localized Injections

Localized Hyal injections are the most common method of Hyal administration (Table S1). Small Hyal aliquots are injected directly into or around the affected area intradermally using a needle or a cannula. Several filler complications can be managed with localized injections, including undesired aesthetic outcomes, the Tyndall effect, small lumps, non-inflammatory nodules, delayed facial edema caused by allergic reactions, and non-emergent vascular complications.

The dosage used in localized injections and the number of interventions depend on the specific complications and the treated area [3]. For instance, a single intralesional injection of 15 IU Hyal combined with corticosteroids was sufficient to resolve delayed hypersensitivity reactions triggered by COVID-19 vaccine after lip filler injections, which caused erythematous swelling [32]. Similarly, periocular edema is typically treated with low-dose Hyal. In a study including 61 patients, 92% resolved the periocular edema with a single treatment of 15–90 IU, injected at 1–3 points, depending on the edema extent, followed by a gentle massage [83]. Interestingly, 10% of patients needed lower eyelid blepharoplasty after Hyal treatment to completely resolve the complication [83]. Blepharoplasty may be necessary even after high-dose Hyal injections if some filler material remains [59]. Indeed, when filler persists, surgical intervention, such as abscess or nodule drainage, may be necessary in combination with single or multiple Hyal treatments [57,106].

High-dose treatments are also commonly chosen in clinical practice. For instance, a single injection with 1650 IU Hyal was sufficient to resolve lip swelling triggered by vaccination after augmentation procedure [53]. Additionally, as outlined by the ACE Group guidelines, localized high-dose Hyal (450–1500 IU) can also be used when vascular complications such as ischemia, necrosis, erythematous swelling, or vision compromise arise, since perivascular Hyal can infiltrate blood vessels and retain its activity longer than intravascular injections [41]. This approach is widely employed in current clinical practice [73,94,95,96,119]. Multiple interventions with either low- or high-dose Hyal are performed when symptoms persist [38,42,99]. Massages and warm compresses are typically applied following Hyal administration to improve its diffusion [22,66,103].

Overall, the implementation of imaging techniques to identify the filler material and ultrasound (US)-guided injections enables precise and controlled Hyal administration, reducing the need for higher doses [12]. Cases in the literature reported a failure in symptom resolution following an initial “blind” high-dose Hyal administration. Following imaging examinations, such as high-frequency ultrasonography (HFUS), US, or magnetic resonance imaging (MRI), the affected area could be precisely identified, and a single low-dose US-guided Hyal injection was then enough to resolve the symptoms [57,63,66,99].

### 6.3. High Dose Pulsed Hyaluronidase and THIS and FAT Techniques

The high-dose pulsed Hyal (HDPH) protocol was developed in 2017 by DeLorenzi to treat vascular occlusion and skin necrosis, consisting of hourly repeated perivascular and intravascular high-dose Hyal injections, followed by warm compresses and massages to promote Hyal diffusion. Several cycles can be performed until ischemia resolution, assessing tissue perfusion every 20 min. Hyal dosage is typically within a range of 450–1500 IU; however, it varies based on the complication severity [120]. The protocol remains widely used in current clinical practice and has proven to be highly effective even if the intervention is delayed [45,116].

Recently, a new protocol “THIS and FAT” has been developed, combining the HDPH approach with administration of a vasodilator agent, antibiotics, platelet-rich fibrin for wound care, debridement of wound surfaces for severe ischemia, and fat harvesting for fat membrane for wound repairing. US and capillary refill time are implemented before and after the treatments to monitor the perfusion. Intravascular Hyal injections are performed under US guidance. This approach is particularly promising, as it outlines specific steps for each of the five clinical stages of ischemia [36].

### 6.4. Intravascular Injections

Although perivascular Hyal injections are effective in resolving vascular occlusion, intravascular administrations are often necessary to minimize the dissolution time and promptly restore blood flow, especially in emergency scenarios [14]. Specifically, super-selective intra-arterial thrombolytic therapy (IATT) is a very efficient strategy for the treatment of vascular embolism. Digital subtraction angiography (DSA) is used to detect flow interruption and to precisely administer high-dose Hyal [44,108,110,111]. Doppler or Duplex US and computed tomography (CT) are also valid tools to identify vessel occlusion [40,107,113]. Particularly, HFUS can effectively identify the filler’s exact location, size, and composition. Indeed, due to their hydrophilic nature, HA fillers appear anechoic, shifting to hypoechoic as the filler is absorbed into the tissue [112]. Consequently, although intravascular injections are commonly performed with high Hyal doses, implementing HFUS-guided treatment enables a more precise Hyal administration, allowing for lower cumulative Hyal doses and better outcomes. A recent study including 21 patients with facial vascular occlusion demonstrated that timely HFUS detection and US-guided injection allowed for resolution after a single low-dose (35–50 IU) treatment [112].

Intravascular intervention may be necessary when previous localized treatments failed, as it often occurs in cases of visual impairment [87]. When severe vascular compromise persists, multiple intravascular treatments may be required to dissolve the filler material [113,114].

Antibiotics and corticosteroids are crucial in preventing infections and inflammation and are commonly administered concomitantly or after Hyal injections. Additionally, intravascular Hyal injections are typically combined with vasodilator or thrombolytic agents to promote blood flow restoration [87,110]. As nitroglycerine patches are no longer recommended [121], papaverine is often used as a vasodilator, due to its ability to promote smooth muscle relaxation and inhibition of calcium ion channels [44,108,122]. In several cases, HBOT sessions have been administered to enhance perfusion [40,85]. RF therapy and local platelet-rich plasma injections have been occasionally reported as strategies to promote tissue repair [44,110].

### 6.5. Targeted Nodule Infiltration

Non-inflammatory nodules can be treated by injecting Hyal into the filler material or in the surrounding area. Massages help distribute Hyal, enhancing filler degradation. Based on the filler properties and nodule size, Hyal dose may vary; however, a low dose is typically used [3]. The Italian consensus recommends a dose of 45–150 IU for nodules up to 1.5 cm [13]. Indeed, a recent case reported that a single bolus of 150 IU Hyal injected at four points into a 1.5 cm diameter nodule was sufficient to quickly dissolve it [43]. However, persistent nodules may require multiple sessions or higher Hyal dose [117]. If no infection is present and the patient wishes to preserve the filler effects, non-inflammatory DONs could also be treated with steroid therapy, without Hyal administration [3].

Conversely, inflammatory nodules and DONs, nodules formed after hypersensitivity reactions or infections, and granulomas usually require higher Hyal doses or repeated injections [55,63,69,98,106]. US or CT examination can be implemented to identify and localize the nodule, enabling targeted treatment delivery [55,63]. A biopsy is often performed to histologically analyze the material within the nodule and can be a valuable tool for detecting infection, foreign body granulomatous reactions, and inflammatory infiltrates, enabling the correct diagnosis and the effective strategy for antibiotic or steroid treatments [55,63,98,115]. Incision and drain or aspiration of material from abscesses and nodules may also be necessary to relieve symptoms and analyze the exudate [58,106]. Notably, inflammatory nodules and granulomas are frequently characterized by the presence of biofilm or thick fibrous capsules surrounding the filler material, resulting from infection or an inflammatory response. Consequently, Hyal injection around the nodule is commonly not sufficient to dissolve it, and treatment infiltration is necessary [48]. Recently, the modified Munhoz-Cavallieri lavage protocol was successfully implemented in cases of filler-induced refractory facial sterile abscesses. The protocol consists of US-guided drainage of the abscess, followed by multiple US-guided lavages with a solution of high-dose Hyal (4500–12,000 IU), lidocaine, and corticosteroids [118].

Although antibiotics and steroids are typically administered in the presence of infectious or inflammatory nodules, conflicting indications persist among different studies regarding whether they should be administered before or after Hyal injections. The ACE Group recommended an initial antibiotic treatment, followed by Hyal and intralesional steroids for managing inflammatory DONs, as Hyal injections may dissolve the biofilm, promoting the spread of the infection [58]. However, first-line systemic antibiotics or steroids might not always lead to symptom improvement or resolution, requiring a combined strategy [58,63,106]. Recent case reports indicate that intralesional high-dose Hyal combined with intralesional or systemic steroids or antibiotics is an effective approach. However, the timing of antibiotic and steroid administration still varies in clinical practice [55,58,63,98,115].

### 6.6. Management of Vision Compromise

In the event of visual compromise, a prompt interruption of the aesthetic procedure and a rapid intervention are critical to avoid permanent damage [3]. The ACE Group outlined key steps and recommendations for practitioners to implement when managing visual impairment. To preserve vision, time is the most critical prognostic factor; retinal perfusion should be restored within 1–1.5 h to lower intraocular pressure. Repeated and prolonged ocular massages with increasing pressure are also recommended, as they are crucial for dissolving the embolus or dislodging it to a more peripheral position. Overall, a prompt referral to a specialist must be immediately provided [123]. Adjunctive therapies have also been described, such as CO_2_ rebreathing and HBOT to enhance perfusion, aspirin to prevent blood clotting, and pharmaceutical therapies to lower blood pressure [3,13,123]. Additionally, Hyal administration is recommended; however, there are currently no standardized procedures, and various approaches are described in the literature with differing levels of success [3,123].

A high heterogeneity in Hyal strategies in current clinical practice is evidenced by various recent reports in the literature. Several studies have documented using localized high-dose Hyal injections at the treatment site or through peribulbar or intraorbital administration. Multiple high-dose treatments are typically required, as a single bolus dose is often ineffective [39,91,104,119]. However, a complete visual resolution is rare and depends greatly on the extent of vascular involvement at the initial stage. A recent case reported full vision recovery in a woman following multiple high-dose Hyal intraorbital and extraorbital injections within a 4 h period [95]. In contrast, permanent vision loss was documented in a patient despite immediate intervention with a subcutaneous Hyal injection, followed by high-dose subcutaneous and peribulbar Hyal administrations combined with anticoagulants and ocular massage [39].

Overall, supraorbital and retrobulbar injections are frequently employed. As retrobulbar injections carry significant risks and the evidence supporting their clinical effectiveness is limited, experts are very cautious in recommending this method, emphasizing that retrobulbar injections should only be performed in emergency scenarios and administered exclusively by experienced practitioners within specialized clinical settings [124]. Conversely, the supraorbital approach does not require specialized training and should be considered if the embolism is superficial. However, these approaches remain not standardized and may present technical challenges, leading to inconsistent outcomes [3,123].

A recent case described a complete vision recovery achieved with multiple supraorbital injections with lower Hyal doses combined with ocular massages and HBOT sessions [38]. Three recent reports described a combined approach, where localized high-dose Hyal is followed by IATT, showing significant symptom improvements but not complete resolution [35,85,87]. First-line DSA-guided IATT with high-dose Hyal and vasodilators has also been described as a successful approach, although it may require additional IATT, localized treatments, or HBOT sessions to achieve satisfactory recovery [40,100,111,114]. Anticoagulants, steroids, and HBOT are often described as efficient adjunct therapies to promote perfusion [38,40,111].

Additionally, when vision compromise occurs and symptoms are suggestive, practitioners should consider the possible presence of cerebral infarction caused by the filler’s retrograde movement into the internal carotid artery [111]. Consequently, MRI and CT scans have been described to investigate intracranial infarction and, ultimately, provide a targeted treatment [37,100,104,111].

## 7. Conclusions

Hyal is the treatment of choice for managing various complications caused by HA dermal fillers during aesthetic procedures. Indeed, its high safety profile and rapid filler degradation ability render Hyal an effective solution in both non-emergent and emergency scenarios. However, when considering Hyal administration, practitioners should be aware of and inform patients about the possibility that, although rare, local adverse events or allergic reactions could occur. Additionally, Hyal treatment can compromise the desired aesthetic results by non-selectively degrading HA, especially when administered in high doses. In non-emergent situations, it is therefore often preferred to use lower doses of Hyal to address the complications without significantly affecting the filler’s cosmetic effect.

Hyal has an immediate effect on HA fillers, making it suitable for both aesthetic corrections and emergency treatments. However, due to its short half-life of about 2 min and activity lasting 24–48 h [3], multiple treatments are often necessary to fully resolve symptoms, particularly with highly cross-linked fillers. New promising strategies, such as HFUS-guided injections, may not be accessible everywhere, and commercial Hyal formulations are not available worldwide. Nevertheless, in countries like Italy, where Hyal formulations have not yet been approved for treating aesthetic complications, galenic formulations are endorsed, given their safety and effectiveness.

As evidenced by the most recent cases reported in the literature over the past five years, the absence of standardized protocols, variable dosage recommendations, and heterogeneous availability of technical resources result in a great variability in clinical practice regarding Hyal doses, injection techniques, and concomitant treatments. Consequently, in their decision-making, practitioners should always consider the filler’s properties, the treated area, the available Hyal formulation, and the specific complication to determine the optimal treatment strategy in each case. However, the increasing number of cases described in the literature where various complications from HA filler procedures have been successfully managed with different Hyal administration strategies is compelling evidence that Hyal remains a safe and effective tool for addressing undesired aesthetic outcomes and filler complications.

Formulating clear guidelines and developing standardized protocols through controlled trials should be the focus of future research. This will support practitioners in their decision-making processes, reducing response time and ensuring consistent outcomes. Moreover, patient-specific dosing algorithms based on imaging and filler properties could be developed to optimize Hyal treatments.

## Data Availability

No new data were created or analyzed in this study. Data sharing is not applicable to this article.

## Supplementary Materials

The following supporting information can be downloaded at: https://www.mdpi.com/article/10.3390/jcm15010279/s1, Table S1: Current management strategies adopting hyaluronidase for the treatment of complications due to HA filler injections, based on case reports published between 2020 and 2025.

## Abbreviations

The following abbreviations are used in this manuscript:

CT	Computed tomography
DON	Delayed-onset nodules
DSA	Digital subtraction angiography
HA	Hyaluronic acid
HBOT	Hyperbaric oxygenation treatment
HDPH	High-dose pulsed hyaluronidase
HFUS	High-frequency ultrasonography
Hyal	Hyaluronidase
IATT	Intra-arterial thrombolytic therapy
IDT	Intradermal test
IU	International unit
MRI	Magnetic resonance imaging
RF	Fractionated radiofrequency
RHH	Recombinant human hyaluronidase
US	Ultrasound
