# Supplementary material for: Hyaluronidase Use in Aesthetic Medicine: Formulations, Safety, and Clinical Practice
Source: J Clin Med. 2025 Dec 30;15(1):279. doi: 10.3390/jcm15010279 (PMC12787120; doi:10.3390/jcm15010279)
Supplement: Supplementary file 1 [file jcm-15-00279-s001.zip › Supplementary Table_ Revised.pdf]

Supplementary Table

Table S1. Current management strategies adopting hyaluronidase for the treatment of complications due to HA filler injections, based on case reports published between 2020 and 2025.

Hyal treatment strategy	Dermal filler complications	Concomitant treatments	Comments and considerations
**Localized injections:**<br><br>**- Single treatments** [28,29,32,33,35,36,46,54,56,58,59,61–83,118]<br><br>**- Multiple treatments** [12,22,23,34,37–39,42,45,53,55,57,60,73,83–106,117–119]	**- Vascular complications and Tyndall effect** [12,22,23,34–39,42,45,46,56,60,61,63–68,70,72–85,87–96,100,102–105,119]<br><br>**- Overfilling, filler misplacement** [57,62,70,71,74,99,101,117]<br><br>**- Unsatisfactory results** [28,59,83]<br><br>**- Nodules or inflammatory reactions** [29,32,33,53–55,58,63,69,97,98,106,117,118]<br><br>**- Vestibular disease** [86]	**- Pre- or post-treatment Ab or steroid therapy** [12,22,23,32–37,39,42,53–55,57,58,61,63,70,70,73,74,74–77,82,86,87,89,91,92,94,97,98,100,102–104,106,118]<br><br>**- HBOT, laser therapy, FR-MN** [34,38,45,56,60,61,65,76,84,85,89,92,97,103,104]<br><br>**- Co-treatment with collagenase, lipase, or hypochlorous acid** [62,67,106]<br><br>**- Surgical intervention** [29,57–59,83,106,118]	**- High dose Hyal** [22,23,36,39,45,53,61,64,65,67,68,73,78,81,84,86,87,91,94–96,102,104,117–119]<br><br>**- Low dose Hyal** [12,32,42,46,66,76,83,90,99,105,106]<br><br>**- US-guided Hyal injections** [12,36,57,62–64,66,71,72,79,89,90,96,97,99,118]<br><br>**- Combined with intravascular injections** [35,39,87,94,100]<br><br>**- MRI, CT, and US** for diagnosis and assessment [37,55,60,70,73,74,74,75,79,86,94,96,99,103–105,119]
**HDPH and "THIS and FAT" protocols** [36,45,116]	**- Vascular complications** [36,45,116]	**- Firm massages and HBOT** [45]<br><br>**- "THIS and FAT" includes:** BTX-A injection, Ab and PRF therapy, debridement and dermabrasion of wound surfaces for ischemia above grade three, and fat harvesting for	**- Multiple sessions until resolution** [45,116]<br><br>**- Compression test and Doppler US** for diagnosis and assessment [36,45]

Hyal treatment strategy	Dermal filler complications	Concomitant treatments	Comments and considerations
		fat membrane to repair ischemic wounds [36]	**- US-guided intra-arterial delivery** for more precision and efficacy [36]
**Intra-arterial or intravascular injections / IATT** [35,36,40,44,85,87,100,107–114]	**- Vascular complications** [35,36,40,44,85,87,100,107–114]	**- Vasodilators or thrombolytic agents** [35,44,87,100,108,110,111,114]<br><br>**- Ab, steroid, or RF-MN therapy** [35,44,87,100,107,108,110–112]<br><br>**- Local platelet-rich plasma injections** [110]<br><br>**- HBOT** [40,85]	**- DSA-guided super-selective IATT using high-dose Hyal** [35,40,44,87,100,108,109,111,114]<br><br>**- US-guided Hyal injections** [36,107,112,113]<br><br>**- Doppler US or CT** for diagnosis [40,107,109,113]<br><br>**- Multiple sessions or additional localized Hyal injections** [36,87,100,113,114]<br><br>**- Intra-arterial Hyal administered after failed localized injections** [35,85,87,107,109]
**Ultrasound-guided:**<br><br>**- Localized injections** [12,36,57,62–64,66,71,72,79,89,90,96,97,99,118]<br><br>**- Intra-arterial injections** [36,107,112,113]	**- Overfilling, residual filler, and filler misplacement** [57,62,71,99]<br><br>**- Vascular complications** [12,36,63,64,66,72,79,89,90,96,107,112,113]<br><br>**- Inflammatory reactions** [97,118]	**- Co-treatment with collagenase and lipase** [62]<br><br>**- Pre- or post-treatment Ab or steroid therapy** [12,36,57,63,89,97,107,112,118]<br><br>**- HBOT, laser therapy, FR-MN** [89,97]	**- Multiple sessions or additional localized Hyal injections** [12,62,90,96,99,113,118]<br><br>**- Additional MRI** to determine the presence of filler material [63,99]<br><br>**- US-guided Hyal administered after failed blind injections** [57,63,97,107]

Hyal treatment strategy	Dermal filler complications	Concomitant treatments	Comments and considerations
**Targeted nodule infiltration** [33,43,55,58,63,69,98,106,115,117,118]	**- Foreign body granulomatous reaction** [63,98,115]<br><br>**- Inflammatory nodules due to hypersensitivity reaction** [55,69,106], **or secondary to COVID-19 infection** [33]<br><br>**- Non-inflammatory nodules** [43,117]<br><br>**- Abscess** [58,118]	**- Pre- or post-treatment Ab or steroid therapy** [33,55,58,63,98,115,118]<br><br>**- Co-treatment with hypochlorous acid** [106]<br><br>**- Surgical intervention** [58,106,118]	**- High-dose Hyal or multiple sessions** [55,58,63,98,115,117,118]<br><br>**- Biopsy or aspirated fluid** for diagnosis [63,98,115,118]<br><br>**- US and CT** for diagnosis [55,118]<br><br>**- Prompt recognition of filler complication** to prevent misdiagnosis [98]<br><br>**- First-line Ab therapy without a confirmed infection** will result in no improvement or worsening and delay of effective treatment [55,58]
**Vision impairment protocols**:<br><br>**- IATT** [35,40,85,87,100,111,114]<br><br>**- Localized injections** [37–39,70,74,77,85,87,91,95,100,104,119]	**- Vision loss** [35,37–39,77,85,87,95,100,111,114]<br><br>**- Ophthalmoplegia** [35,70,74,77,104,119]<br><br>**- Visual compromise without vision loss** [40,91]<br><br>**- Concomitant cerebral infarction** [37,95,111]	**- Vasodilators** [35,37,74,87,100,111]<br><br>**- Post-treatment Ab, steroid, or anticoagulant therapy** [35,37,39,70,74,77,87,91,100,104,111,114]<br><br>**- HBOT** [38,40,85]	**- DSA-guided super-selective IATT using high-dose Hyal** [35,40,85,87,100,111,114]<br><br>**- Multiple sessions** [35,37–39,77,85,87,91,95,100,114,119]<br><br>**- MRI or CT** for diagnosis or investigation of cranial infarction [37,39,40,70,74,77,95,100,104,111,119]

Vascular complications include vascular embolism, vascular occlusion, edema, ischemia, tissue necrosis, ecchymosis, vision impairment, alopecia, and filler compression of vessels. Abbreviations: Ab, antibiotic; BTX-A, botulinum toxin type A; CT, computed tomography; DSA, digital subtraction angiography; HBOT, hyperbaric oxygen therapy; HDPH, high-dose pulsed Hyal technique; Hyal, hyaluronidase; IATT, intra-arterial thrombolytic therapy; RF-MN, radiofrequency microneedling; MRI, magnetic resonance imaging; IU, units; US, ultrasound.
